# Measurement, Analysis and Interpretation of Pressure/Flow Waves in Blood Vessels

**DOI:** 10.3389/fphys.2020.01085

**Published:** 2020-08-27

**Authors:** Jonathan P. Mynard, Avinash Kondiboyina, Remi Kowalski, Michael M. H. Cheung, Joseph J. Smolich

**Affiliations:** ^1^Heart Research, Murdoch Children’s Research Institute, Melbourne, VIC, Australia; ^2^Department of Paediatrics, The University of Melbourne, Melbourne, VIC, Australia; ^3^Department of Biomedical Engineering, The University of Melbourne, Melbourne, VIC, Australia; ^4^Department of Cardiology, The Royal Children’s Hospital, Parkville, VIC, Australia

**Keywords:** haemodynamics, pulse wave analysis, wave intensity analysis, wave separation, wave speed, reservoir pressure

## Abstract

The optimal performance of the cardiovascular system, as well as the break-down of this performance with disease, both involve complex biomechanical interactions between the heart, conduit vascular networks and microvascular beds. ‘Wave analysis’ refers to a group of techniques that provide valuable insight into these interactions by scrutinizing the shape of blood pressure and flow/velocity waveforms. The aim of this review paper is to provide a comprehensive introduction to wave analysis, with a focus on key concepts and practical application rather than mathematical derivations. We begin with an overview of invasive and non-invasive measurement techniques that can be used to obtain the signals required for wave analysis. We then review the most widely used wave analysis techniques—pulse wave analysis, wave separation and wave intensity analysis—and associated methods for estimating local wave speed or characteristic impedance that are required for decomposing waveforms into forward and backward wave components. This is followed by a discussion of the biomechanical phenomena that generate waves and the processes that modulate wave amplitude, both of which are critical for interpreting measured wave patterns. Finally, we provide a brief update on several emerging techniques/concepts in the wave analysis field, namely wave potential and the reservoir-excess pressure approach.

## Introduction

Cardiovascular disease has a profound impact on people around the world and across the human lifespan, accounting for 31% of all deaths globally (World Health Organization, 2013), as well as being the most common and costly category of birth defects (Centers for Disease Prevention and Control, 2019). While many factors contribute to the incidence and progression of cardiovascular disease, adverse outcomes are ultimately determined by a failure or ineffectiveness of the biomechanical system to deliver oxygenated blood to organs and tissues. Importantly, the major biomechanical properties of the heart and circulatory system, including cardiac contraction, ventriculo-vascular coupling, large artery stiffness, and microvasculature properties, all influence the pattern of pressure/flow waves that can be measured in blood vessels. The chief aim of ‘wave analysis’ is therefore to reliably measure and then ‘decode’ the pattern of waves to uncover insights about disease processes and therapies that may not be provided by conventional metrics such as systolic and diastolic blood pressure.

A range of established and emerging wave analyses are available, including pulse wave analysis, wave separation, wave intensity (with several variations), and reservoir-excess pressure analysis. While each has strengths and weaknesses, along with aspects that are still being debated and refined, a number of prominent clinical studies have shown that wave analyses provide valuable prognostic information over and above traditional risk factors (Manisty et al., 2010; Weber et al., 2010, 2012; Davies et al., 2014; Zamani et al., 2014; Narayan et al., 2015; Zamani et al., 2016; Chirinos, 2017; Chiesa et al., 2019). However, to optimize prognostic value and to obtain mechanistic insights while avoiding misinterpretations, it is essential to develop a clear understanding of the physiological determinants of the various indices obtained from wave analysis.

The aim of this review is to introduce key techniques and concepts relating to arterial wave analyses, whilst also providing an update on recent developments and emerging techniques in the field. We start by briefly reviewing techniques for measuring blood pressure, flow, and velocity waveforms, then cover the most well-established wave analysis techniques (pulse wave analysis, wave separation and wave intensity analysis). We then review the biomechanical factors that generate and modulate pressure/flow waves, an understanding of which is likely to aid in the interpretation of wave patterns. Finally, several emerging concepts in the wave analysis field are briefly discussed (reservoir and excess pressure analysis, and wave potential). To appeal to a broad readership, this review will contain minimal mathematics and no derivations, but references will be provided where such details can be found.

## What Is a Wave?

**FIGURE 1:**
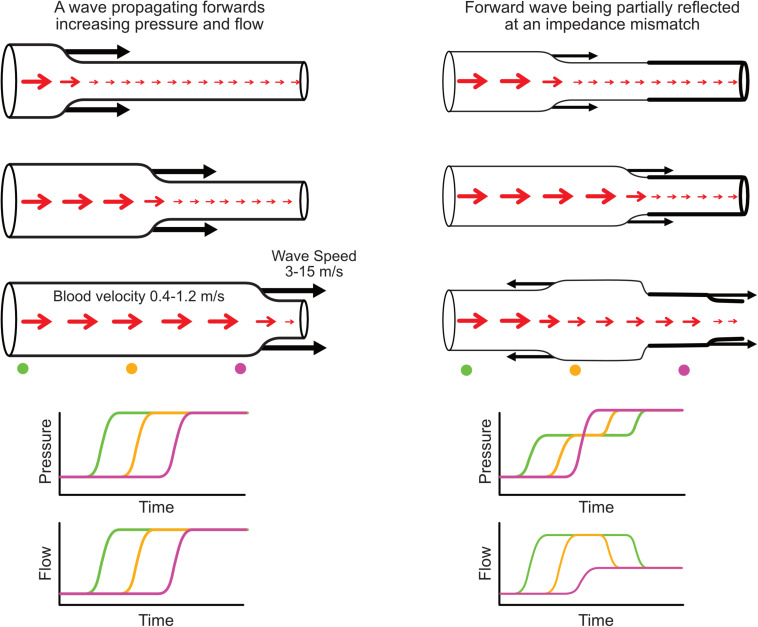
**(Left)** Illustration of a forward-propagating wave that increases pressure and flow. **(Right)** A forward-propagating wave encounters an impedance mismatch (increased vessel stiffness indicated by thicker wall) and is partially transmitted and partially reflected. The reflected wave propagates backward and causes a further increase in pressure but a decreased flow. Red arrows indicate blood flow or average blood velocity over a cross-section (larger arrow means higher flow/velocity), while black arrows indicate wave speed and direction of wave propagation. Increased pressure is indicated by increased vessel diameter. Time plots (bottom) indicate the evolution of pressure and flow at three locations along the vessel indicated with color-coded circles.

While the term ‘wave’ is attributed different meanings in different settings, in this review we adopt the definition of Hughes et al. (2008) that a *wave is a change in pressure and flow that propagates along a blood vessel* (Figure 1). Conversely the term ‘waveform’ herein refers to the pressure or flow pulse signal that can be measured at a particular vascular location. Waves travel at a velocity, known as *wave speed* or *pulse wave velocity*, that is typically more than ten times faster than the velocity of flowing blood; for example, pulse wave velocity of the aorta is around 5 m/s in youth, although this increases more than twofold over the normal human lifespan (McEniery et al., 2005). Every wave has an effect on both pressure and flow, and these effects are intrinsically linked by the *characteristic impedance* of the vessel, which in large arteries is proportional to wave speed and inversely proportional to vessel cross-sectional area. When a wave encounters a change in characteristic impedance, some of the wave energy is reflected and some is transmitted (Figure 1).

Pressure and flow waveforms result from a set of waves that pass by the measurement location, with each wave causing an increment or decrement in those waveforms (Figure 1, bottom panels). By performing wave analysis, we aim to extract information about the waves that produced the pulse waveforms and make inferences about their origins. For example, common questions in the field of arterial haemodynamics include: To what extent do reflected waves contribute to rises in blood pressure with aging or in certain disease conditions? Where are wave reflections occurring and does the distance to the reflection site (or ‘effective distance’ given that there are many reflection sites) change with age or disease? How much does wave reflection versus aortic characteristic impedance contribute to left ventricular afterload? Which specific waves are increasing or decreasing with an intervention and what insight does this provide about mechanisms of action? In coronary arteries, what is the interplay between upstream (ventriculo-aortic) vs. downstream (intramyocardial) forces in generating and modulating myocardial perfusion?

A fundamental precursor to addressing such questions is ensuring that the required physiological data can be measured, and with adequate quality. Depending on available equipment, resources and expertise, this is not always easy in a clinical setting. Most wave analyses require a pressure waveform (or a distension-based surrogate) and a flow or velocity waveform, although some techniques use only pressure. Importantly, since wave information resides in the shape of the waveform(s), the reliability of the final analysis depends on acquiring reproducible signals with high fidelity.

## Measurement of Pressure Waveforms

### Invasive Measurement

The gold-standard for acquiring the blood pressure waveform is invasive measurement, either with a micromanometer-tipped catheter or fluid-filled catheter and external manometer (Papaioannou et al., 2009; Nichols and O’Rourke, 2011). Micromanometer-tipped catheters provide a high fidelity waveform due to their excellent frequency response. While often used in animal studies (van den Bos et al., 1982; Khir and Parker, 2005; Penny et al., 2008), similar single-use pressure wires are used in humans, but are expensive. Fluid-filled catheter systems are cheaper, but their frequency response can be poor and should be tested to ensure waveform features are faithfully captured (Nichols and O’Rourke, 2011). Even in a system with an adequate frequency response, damping can develop over time, for example, if the catheter is not properly flushed. While investigators should be vigilant against such damping, Howard et al. (2019) recently described an artificial intelligence approach that may help automatically identify this issue.

### Non-invasive Measurement

Applanation tonometry and the volume-clamp method are non-invasive techniques for measuring the arterial pressure pulse waveform. Applanation tonometry involves ‘applanating’ (slightly compressing against bone) a superficial artery with a pen-like pressure transducer (Drzewiecki et al., 1983; Kelly, 1989), which requires good dexterity and experience, but is relatively easy to learn. Dependence on a manual operator is potentially avoidable with emerging wearable devices (Garcia-Ortiz et al., 2012), although attaining and maintaining correct sensor position is a key challenge. Adequate applanation can be achieved in most individuals for the radial artery, but can be more difficult in other locations (e.g., brachial or carotid arteries) (Nichols and O’Rourke, 2011). Calibration of the pulse waveform is generally performed with conventional brachial cuff pressures, which may involve errors due to cuff pressure inaccuracies and pulse amplification from brachial to radial sites (Verbeke et al., 2005; Picone et al., 2017).

The volume-clamp method, originally described by Peñáz (1973) and refined and commercialized as the Finapres by Wesseling (1995) involves a finger cuff whose air pressure is controlled by a servo that clamps the volume of finger arteries using an infrared photoplethysmography (PPG) signal. This method provides high fidelity waveforms, is often used where continuous non-invasive pressure monitoring is required, and has the benefit of self-calibration, although the precision of absolute pressure values does not meet the AAMI requirement of an error standard deviation less than 8 mmHg (Kim et al., 2014). The technique is widely applicable, but may be difficult to perform in small children or where peripheral vasoconstriction (e.g., due to ‘feeling cold’) lead to an inadequate PPG signal, while accuracy may be reduced if the finger cuff is applied too loosely (Imholz et al., 1998). Compared with the volume-clamp method, tonometry is less cumbersome, involves less discomfort, and can be applied to a range of arterial sites, which may underlie its more widespread use in the wave analysis field.

### Surrogate Measures of the Pressure Waveform

While catheterization, applanation tonometry, and the volume-clamp method in principle measure the actual blood pressure waveform, the techniques discussed below are surrogates, in that a form of distension (diameter, area, or volume change) is used to approximate the pressure waveform after appropriate calibration. When adopting these techniques, two biomechanical factors should be kept in mind. First, the relationship between pressure and distension is not strictly linear. For example, although the carotid pressure-distension relationship can appear approximately linear (Watanabe et al., 1999; Sugawara et al., 2000), Vermeersch et al. (2008) found that a non-linear (exponential) calibration of diameter to pressure improved the waveform fit (RMSE) by 28%. The degree of non-linearity also increases with age and with greater range of distension (Pagani et al., 1979; Langewouters et al., 1984; Meinders and Hoeks, 2004). Second, the pressure-distension relationship exhibits hysteresis due to the viscoelastic properties of vessel walls. This means that although pressure and distension follow a similar trajectory on the ascending limb of the pulse, these signals diverge on the descending (diastolic) limb due to viscous energy losses in the arterial wall (Armentano et al., 1995; Watanabe et al., 1999). Using a computational model, Kang et al. (2019) studied the impact of non-linearities and viscoelasticity on distension-based wave intensity analysis and concluded that the use of diameter as a surrogate for pressure is likely to introduce tolerable errors (<10%), although this requires further confirmation *in vivo*.

**FIGURE 2:**
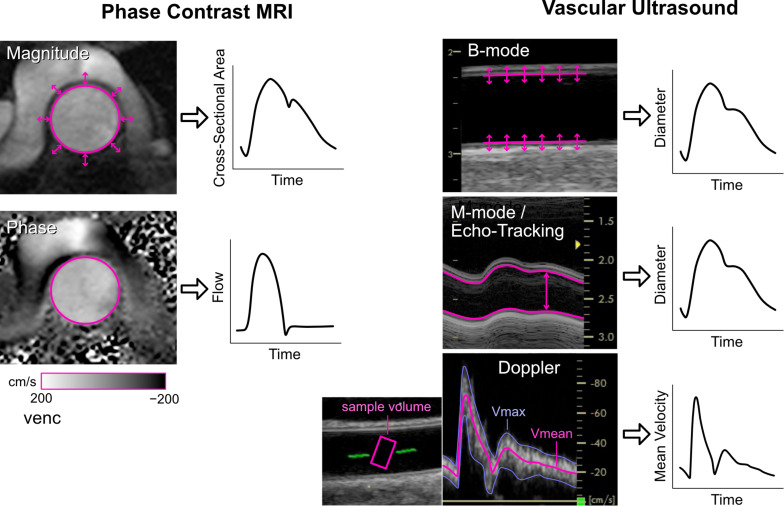
Imaging methods for obtaining cross-sectional area or diameter waveforms and flow or mean velocity waveforms. See text for explanation. Note that echo-tracking is similar to standard M-mode but uses raw radio-frequency signals. For Doppler ultrasound, Vmax and Vmean are the maximum and mean velocities in the sample volume, respectively.

Arterial diameter changes can be measured with a range of techniques (Figure 2). Radio frequency (RF) echo-tracking is considered the most accurate and has high temporal and spatial resolution, but requires specialized equipment and software not available on most standard ultrasound systems (Hoeks et al., 1990; Niki et al., 2002; Segers et al., 2004); results may also be system-dependent (Palombo et al., 2012). Edge-tracking of the arterial wall in B-mode ultrasound images is a lower resolution, but widely available alternative to RF echo-tracking that has acceptable accuracy for total distension, although accuracy of waveform shape has not been investigated (Steinbuch et al., 2016; Di Lascio et al., 2018). M-mode imaging has high temporal resolution and only requires segmentation of one image, in contrast to the lower resolution and many image frames in B-mode (Kowalski et al., 2019a, b). A number of ambulatory sensor technologies are emerging for measurement of arterial distension, such as RF-echo tracking with a flexible ultrasonic sensor array patch (Wang et al., 2018) and low-frequency (∼1 GHz) continuous-wave radar (Buxi et al., 2017; Pisa et al., 2018). High-frequency (∼60 GHz) radar (Johnson et al., 2019) and double integration of an accelerometric signal (Di Lascio et al., 2018) are also showing promising results, despite measuring the motion of the skin surface due to the pulse rather than arterial diameter *per se*.

Aortic cross-sectional area changes have been used as a surrogate of the central pressure waveform and can be obtained via phase contrast magnetic resonance imaging (MRI, Figure 2) (Biglino et al., 2012; Quail et al., 2014; Li et al., 2019). This approach has limited temporal resolution (currently up to ∼100 Hz) and requires segmentation of the aortic circumference from the magnitude images, which can be challenging during low-flow phases of the cardiac cycle or in low-flow segments of the vessel wall that display poor contrast. However, the advantage is that the phase images, obtained with the same MRI sequence, provide simultaneous flow information.

While arterial volume cannot be easily measured directly, volume plethysmography of a limb or finger segment is commonly used to derive a pulse waveform. When a standard arm cuff is inflated and held at a fixed pressure, brachial arterial pulsations induce oscillations in cuff air pressure that can be registered by a device. Supra-systolic oscillometry, which causes arterial occlusion, is employed by a number of devices (Horvath et al., 2010; Lin et al., 2012; Park et al., 2014; Stoner et al., 2014; Bhuva et al., 2019); however, Butlin et al. (2012) reported that this technique led to artifacts such as elimination of the diastolic portion of the waveform. Sub-diastolic oscillometry is non-occlusive and involves less patient discomfort, and is commonly used in commercial devices (Wassertheurer et al., 2010; Butlin et al., 2012; Luzardo et al., 2012; Parikh et al., 2016). Pulsations can also be monitored in the finger with standard photoplethysmography and converted to a pressure pulse with a transfer function (Millasseau et al., 2000).

### Mathematical Derivation of the Central Pulse Waveform

Central aortic blood pressure is of particular interest in many studies, as it represents the direct pressure load faced by the ejecting left ventricle. Although many of the aforementioned techniques acquire peripheral arterial signals, a central aortic pressure waveform can be derived from these via a generalized transfer function or other model-based methods (Chen et al., 1997; Karamanoglu and Feneley, 1997; Gallagher et al., 2004; Wassertheurer et al., 2010; Stok et al., 2011). This is a popular approach for estimation of both central systolic pressure and central wave indices such as augmentation index; however, a number of invasive validation studies have found that central wave indices derived using these methods may have limited accuracy, most likely due to individual variability in the higher frequency components (Chen et al., 1997; Fetics et al., 1999; Millasseau et al., 2003). This limitation does not appear to preclude this approach from having substantive clinical value when using high fidelity applanation tonometry (Weber et al., 2004, 2010; Vlachopoulos et al., 2010; Janner et al., 2013; Zamani et al., 2014), although more evidence is needed for commercial devices that employ surrogate measures of the pressure waveform, such as cuff volume plethysmography, which have considerably lower fidelity.

## Measurement of Flow and Velocity Waveforms

Some wave analysis techniques employ only a pressure waveform (as will be discussed later), but these involve more assumptions and are considered less reliable than techniques that harness blood pressure *and* flow/velocity information (Townsend et al., 2015). Blood flow and blood velocity are distinct physical quantities that both involve challenges in accurate measurement. Blood flow refers to the volumetric transport of fluid (units of volume/time) and is defined over a given arterial cross-section. On the other hand, blood velocity (or ‘flow-velocity,’ the velocity of flowing blood) refers to the speed of a moving particle of blood (units of distance/time). Velocity therefore varies over an arterial cross-section, producing a ‘velocity profile.’ In a long cylindrical tube with constant flow, the velocity profile has a parabolic shape, but more complex velocity profiles arise in real arteries, which are curved and exhibit pulsatile flow (Sigovan et al., 2011; Mynard and Steinman, 2013).

### Invasive Measurement

Perivascular flow probes using transit-time ultrasound methods are considered the gold-standard for invasive flow measurement, although their ±5–15% absolute accuracy is modest (Laustsen et al., 1996; Beldi et al., 2000; Zhang et al., 2019). Commonly used in animal or benchtop experiments (Khir and Parker, 2002; Penny et al., 2008), these probes require good acoustic coupling (e.g., with ultrasound gel) between the vessel wall and internal probe housing, but can maintain accuracy for several years in chronically instrumented experiments (Picker et al., 2000).

In humans, flow probe use tends to be limited to the clinical assessment of bypass grafts (Thuijs et al., 2019), whereas invasive arterial wave analysis tends to be performed based on blood velocity measurements from Doppler ‘flow-wires’ (which actually measure velocity, not flow), such as the Philips Volcano ComboWire that also incorporates a micromanometer for concurrent pressure measurement. This approach has been particularly useful for wave analyses in coronary arteries (Hadjiloizou et al., 2008; Rolandi et al., 2012; Rivolo et al., 2016), but has also been used in the aorta and pulmonary artery (Hughes and Parker, 2009; Lau et al., 2014).

### Non-invasive Measurement

The gold-standard non-invasive method for measuring flow is phase contrast (PC-)MRI, in which the motion of magnetic spins through a magnetic field gradient enables velocity encoding in a specified direction (Pelc et al., 1991). Arterial flow is obtained by setting a 2D acquisition plane through the chosen vessel cross-section, encoding velocity through-plane, and integrating velocities over the arterial cross-section (Figure 2). Signal-to-noise ratio is determined by the encoding velocity (VENC, i.e., the maximum encoded velocity), which must be set appropriately to avoid aliasing (if set too low) or insufficient contrast (if set too high). While standard 2D PC-MRI for clinical flow assessments typically acquire ∼20 frames per cardiac cycle (∼50 ms resolution), this is insufficient to capture high frequency information, such as flow acceleration, required for wave intensity analysis. Higher temporal resolution (∼9 ms) can be obtained with techniques such as spiral SENSE PC-MRI (Steeden et al., 2011; Biglino et al., 2012) or spoiled gradient echo PC-MRI (Bhuva et al., 2019), and even down to 4 ms resolution with retrospectively gated compressed sensing PC-MRI (Peper et al., 2018).

Whereas MRI is relatively expensive and most suited to imaging central vessels, Doppler ultrasound is inexpensive and more suited to imaging peripheral arteries. With pulsed Doppler, a velocity spectrum is acquired over time inside a sample volume that is positioned by the operator, normally in the vessel center and covering at least two thirds of the vessel diameter (Figure 2). The intensities of pixels in each vertical line of the spectrum essentially represent a histogram of velocities within the sample volume at that time point. The spectrum can therefore provide an indication of whether the velocity profile is relatively flat (narrow spectrum) or contains a range of velocities due to a more parabolic, skewed or turbulent profile (broad spectrum).

Software included in most commercial systems allows velocity waveforms to be extracted by tracing the top (or envelope) of the velocity spectrum. However, it is important to recognize that the envelope represents the peak velocity within the sample volume, not the cross-sectional mean velocity required for arterial wave analyses. Several techniques have been described to extract the mean velocity waveform, such as averaging the Doppler spectrum (Niki et al., 2002; Borlotti et al., 2012), or with multi-gate ultrasound systems, color Doppler, or 3D ultrasound (Hoskins, 2011). Regardless of the technique used, various sources of error affect accuracy, including incomplete vessel coverage by the sample volume, non-uniform beam insonation, inappropriate gain settings, inaccurate sample volume placement, imprecise angle correction, spectral broadening, the presence of secondary flow, and operator dependence (Winkler et al., 1995; Mikkonen et al., 1996; Corriveau and Johnston, 2004; Hoskins, 2011; Mynard and Steinman, 2013). Given the many possible sources of error, Kowalski et al. (2017) described a technique that enables correction of any arbitrary scaling of velocity waveforms to obtain mean velocities, which requires accompanying measurements of arterial distention waveforms at the same site, as well as standard blood pressure. While such corrections will not affect the pattern of waves in an individual, they may reduce variability in group analyses and improve sensitivity in clinical studies.

Having provided an overview of techniques for measuring pressure and flow/velocity waveforms, the following sections review the most commonly applied techniques for analyzing these waveforms.

## Pulse Wave Analysis

**FIGURE 3:**
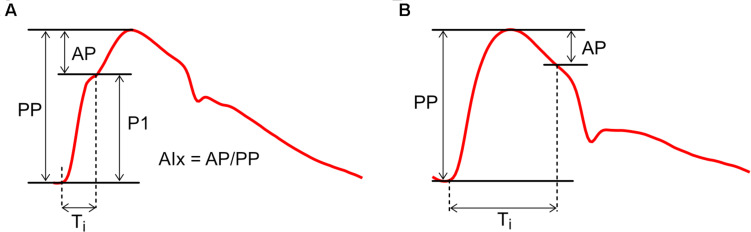
Calculation of Augmentation Index (AIx) with examples of **(A)** positive augmentation and **(B)** negative augmentation. AP, augmentation pressure; P1, initial pressure rise; PP, pulse pressure; *T*_i_, inflection time.

Characterizing blood pressure with only two extreme values (systolic and diastolic) neglects the wealth of information that is present in the shape of the pressure waveform (Figure 3). With pulse wave analysis, characteristic features of the pressure waveform are extracted and inferences about wave dynamics are made on the basis of theoretical (i.e., model-based) links with functional properties of the cardiovascular system. A comprehensive historical review of pulse wave analysis is available in the classic textbook McDonald’s Blood Flow in Arteries by Nichols and O’Rourke (2011). We here focus on *central* pulse wave analysis and the three indices that are most commonly used: augmentation pressure, augmentation index and inflection time.

Pulse wave analysis was founded on the observation that aortic pressure waveforms generally exhibit a characteristic inflection point during systole (Figure 3A). The initial pressure rise up to this inflection point (P1) is thought to relate mainly to the incident or forward-traveling pressure wave (the pressure arising from the natural outflow of the ventricle), while the secondary pressure rise (or ‘augmentation pressure,’ AP) after the inflection point is thought to arise mainly from wave reflection (Kelly et al., 1989). Augmentation Index (AIx), calculated as AP/PP (where PP is pulse pressure), is therefore a surrogate index of arterial wave reflection, while augmentation pressure, if positive, is an ‘additional’ pressure arising from this reflection. In addition, the time from the onset of P1 to the inflection point (*T*_i_) has been used to quantify the total transit time for waves traveling from the ventricle to an effective reflection site and back to the ventricle (Baksi et al., 2009; Sugawara et al., 2010).

While this picture may seem straightforward, a range of studies have suggested that AIx is determined by a complex combination of biomechanical interactions. AIx demonstrates a strong dependence on heart rate and is therefore commonly expressed in a heart-rate-corrected form (AIx@75 bpm) (Wilkinson et al., 2000; Weber et al., 2004). AIx is also modulated by height and differs between males and females, whereas wave separation analysis suggests wave reflection magnitude does not depend on these factors (Hughes et al., 2013). In addition, although AIx increases with age, it plateaus and may even decrease around middle age (Kelly et al., 1989; Mitchell et al., 2004; Fantin et al., 2006); it has been argued that this plateau does not necessarily mean that wave reflection is decreasing, but is a result of mathematical division between two linearly increasing curves (AP and PP) (Namasivayam et al., 2010). Davies et al. (2010) concluded that systolic augmentation was mainly related to reservoir pressure, with only a minor contribution from reflected waves; however, this interpretation now appears moot because current (revised) views acknowledge that wave reflection produces the reservoir pressure (Hughes and Parker, 2020). Nevertheless, AIx has also been shown to depend on ventricular outflow patterns (Karamanoglu and Feneley, 1999), preload (van de Velde et al., 2017), contractility/relaxation properties (Cheng et al., 2012) and forward waves (Fok et al., 2014), although these dependencies may relate in part to re-reflection of backward-traveling waves when they return to the ventricle (Phan et al., 2016a). AIx is negative in some (generally younger) individuals (Figure 3B), but this does not imply the presence of negative wave reflection (Hughes et al., 2013).

Given the many factors that influence AIx, it is tempting to question whether wave reflection is indeed primarily responsible for systolic augmentation. However, a key principle of fluid dynamics in elastic tubes is that, in the absence of wave reflection, pressure and flow waveforms will be identical, albeit scaled by characteristic impedance. Any difference between these waveforms must therefore arise from wave reflection (Parker, 2009; Westerhof and Westerhof, 2017). We therefore agree with the summary of O’Rourke and Mancia (1999) that “augmentation is a manifestation, not a measure of early wave reflection.” Thus, while AIx may be a very useful marker of cardiovascular risk (Nürnberger et al., 2002; Weber et al., 2004, 2010), its limitations in specifically quantifying the biomechanical phenomenon of wave reflection should not be overlooked.

As with AIx, the interpretation of *T*_i_ has been controversial. Although the dominant view has been that reflected waves return to the heart earlier as aging progresses (due to increasing aortic pulse wave velocity), a meta-analysis by Baksi et al. (2009) found that *T*_i_ does not decrease much with age. Based on a modest decrease in *T*_i_ but large increase in aortic pulse wave velocity, some investigators have concluded that the distance to the effective reflection site (*L*_eff_) increases with age (Mitchell et al., 2010; Sugawara et al., 2010). However, the reliability of using *T*_i_ to quantify reflected wave transit time has been questioned; when calculated via wave separation analysis, arrival time of the reflected wave decreased substantially and indicated a decreasing or invariant *L*_eff_ with advancing age (Segers et al., 2007; Phan et al., 2016b). Lastly, *T*_i_ is also affected by the shape of the waveform and reliable identification of the inflection point is not always possible (Nichols and O’Rourke, 2009; Phan et al., 2016b), with modeling data suggesting that *T*_i_ may not be an accurate measure of reflected wave return time (Westerhof and Westerhof, 2012). Thus, inferences about arterial biomechanics based on *T*_i_ should be treated with caution.

## Wave Separation

**FIGURE 4:**
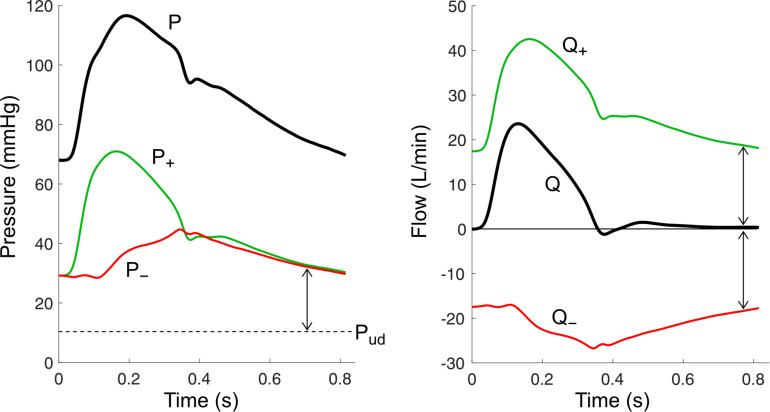
Wave separation analysis for pressure (P) and flow (Q) signals. Forward components (P_+_ and Q_+_) and backward components (P_–_ and Q_–_) are calculated using the equations in Table 1, with an assumed undisturbed pressure (*P*_ud_) of 10 mmHg. Arrows indicate wave potential at that time.

With pulse wave analysis, information about forward (incident) and backward (reflected) waves are estimated from the shape of the pressure waveform. However, as discussed above, various limitations make this approach non-ideal. Wave separation is therefore considered the ‘gold-standard’ method for investigating wave phenomena, since information about forward and backward waves are specifically quantified by decomposing the pressure waveform (*P*) into two separate signals using principles of fluid dynamics in compliant tubes. The forward component of pressure (*P*_+_) represents the contribution of forward waves to measured pressure, while the backward component of pressure (*P*_–_) represents the contribution of backward waves (Figure 4). The caveat is that wave separation is agnostic to the mechanisms that give rise to forward or backward waves; for example, it cannot be used to determine whether an increase in ascending aortic *P*_+_ is caused by a forward wave generated by active ventricular contraction or by passive reflection of a backward wave when it reaches the aortic valve. Such conclusions must be inferred through other means (model-based or experimental inferences).

Pressure wave separation, originally described by Westerhof et al. (1972), can be achieved with the following simple equation when *P* is accompanied by a measured flow waveform (*Q*),

(1) $$P_{\pm }=\frac{1}{2}\left(P-P_{\text{ud}}\pm Z_{\text{c}}Q\right)$$

To apply this formula, two parameters must be estimated. The first is undisturbed pressure (*P*_ud_) and will be discussed later in the *Wave Potential* section; briefly, *P*_ud_ endows physical meaning to the absolute values of *P*_±_, whereas if this parameter is optionally not included, only changes in *P*_±_ are meaningful (Mynard and Smolich, 2014b). The second, characteristic impedance (*Z*_c_), has an important physical meaning that can be easily understood via a fundamental equation of fluid dynamics in compliant tubes called the water hammer equation. This states that incremental changes in pressure and flow arising from forward waves (*dP*_+_ and *dQ*_+_) are intrinsically linked via

(2) $$dP_{+}=Z_{\text{c}}dQ_{+}$$

This means that a pressure wave (a propagating pressure change) is always accompanied by a corresponding flow wave (propagating flow change) and vice versa. Moreover, Equation (2) shows that in a situation where only forward waves exist, measured pressure and flow waveforms will have an identical shape and their ratio will be equal to *Z*_c_. Characteristic impedance in large vessels is determined by wave speed (*c*, i.e., local pulse wave velocity) and cross-sectional area (*A*) via

(3) $$Z_{\text{c}}=\frac{\rho c}{A}$$

where ρ is blood density. Considering Equation (1), we see that an increase in vessel stiffness (which increases wave speed) or a decrease in vessel area will cause a larger pressure change for a given flow change. This has potentially crucial implications for explaining changes in blood pressure with advancing age. For example, a threefold increase in ascending aortic wave speed (Redheuil et al., 2010) would be expected to cause a corresponding threefold increase in the forward component of pressure (e.g., from 30 to 90 mmHg), all other factors being equal. Importantly, however, aortic cross-sectional area also increases with age, offsetting the effect of increased wave speed; the extent of this offsetting effect remains unclear, however, given conflicting data in the literature (Mitchell et al., 2004; O’Rourke and Nichols, 2005; Hickson et al., 2010; Redheuil et al., 2011; Devos et al., 2015).

Another key concept in wave separation analysis is that forward waves cause pressure and flow to change in the same direction, whereas backward waves cause pressure and flow to change in opposite directions. For example, a forward wave that increases pressure will also increase flow, whereas a backward wave that increases pressure will have a flow-decreasing effect, noting that the water hammer equation for backward waves has a negative sign (*dP*_–_ = −*Z*_c_*dQ*_–_). This principle forms the basis for the notion that wave reflection must ultimately be responsible for *any* differences in the shape of arterial pressure and flow waveforms (Westerhof et al., 2010; Nichols and O’Rourke, 2011).

While we have discussed wave separation for pressure, a similar approach can also be applied to calculate forward and backward components of flow (Westerhof et al., 1972), velocity (Parker, 2009), diameter or area distension (Feng and Khir, 2010; Biglino et al., 2012), wave intensity (Parker and Jones, 1990), wave power, and hydraulic power (Mynard and Smolich, 2016). As can be seen in Table 1, all of these calculations rely on the estimation of local characteristic impedance or wave speed.

**TABLE 1:** Variations of wave intensity/power and wave separation.

Signals	Wave intensity (Units)	Wave separation	References
		*P*_±_ = [*P*−*P*_ud_±ρ*cdU*]/2	
Pressure-velocity	*dI=dPdU* (W/m^2^)	*U*_±_ = [*U*±(*P*−*P*_ud_)/(ρ*c*)]/2	Parker and Jones, 1990
		*dI*_±_ = ±[*dP*±ρ*cdU*]^2^/(4ρ*c*)	
Diameter-velocity	*dI*^D^ = *dDdU* (m^2^/s)	$\begin{array}{c} D_{\pm }=\left[D-D_{\text{ud}}\pm DU/\left(2c\right)\right]/2\\ U_{\pm }=\left[U\pm 2c\ln\left(D/D_{\text{ud}}\right)\right]/2\\ dI_{\pm }^{\text{D}}=\pm c{\left[dD\pm DdU/\left(2c\right)\right]}^{2}/\left(2D\right) \end{array}$	Feng and Khir, 2010
Area-velocity	*dI*^A^ = *d*(*ln*⁡*A*)*dU* (m/s)	$\begin{array}{c} \ln A_{\pm }=\left[\ln\left(A/A_{\text{ud}}\right)\pm U/c\right]/2\\ U_{\pm }=\left[U\pm c\ln\left(A/A_{\text{ud}}\right)\right]/2\\ dI_{\pm }^{\text{A}}=\pm c{\left[d\ln A\pm dU/c\right]}^{2}/4 \end{array}$	Biglino et al., 2012

**Signals**	**Wave power (units)**	**Wave separation**	**References**

		*P*_±_ = [*P*−*P*_ud_±*Z*_c_*Q*]/2	
Pressure-flow	*d*π = *dPdQ* (W)	*Q*_±_ = [*Q*±(*P*−*P*_ud_)/*Z*_c_]/2	Mynard and Smolich, 2016
		*d*π± = ±(*dP*±*Z*_c_*dQ*)^2^/(4*Z*_c_)	
		$\Pi_{\text{P±}}=\left[2PQ\pm Z_{\text{c}}Q^{2}\pm \left(P^{2}-P_{\text{ud}}^{2}\right)/Z_{\text{c}}\right]/4$	

*Note that (1) the wave intensity surrogates involving diameter (dI^D^) or cross-sectional area (dI^A^) instead of pressure (dI) do not have units of intensity; (2) except for pressure-flow, expressions for wave separation have been modified from the original references to incorporate wave potential by introducing an undisturbed pressure (P_ud_), diameter (D_ud_) or area (A_ud_) using derivations analogous to those for pressure-flow (Mynard and Smolich, 2014b); (3) hydraulic pressure power (Π_P_ = PQ) may also be separated into forward and backward components (Π_P__±_).*

### Characteristic Impedance and Wave Speed

As discussed above, characteristic impedance (*Z*_c_) represents the intrinsic relationship between pressure and flow when waves are traveling in one direction only. It can be estimated from measured pressure and flow signals, which historically has been achieved in the frequency domain (via Fourier transform) by calculating the average ratio of pressure and flow harmonics in a certain frequency range (O’Rourke and Taylor, 1967). The range may include only high frequency harmonics (e.g., 15–25 Hz) or a lower frequency range (e.g., 5–15 Hz), but the result appears to be relatively insensitive to the exact range chosen (Dujardin and Stone, 1981). A simpler time domain method was proposed by Dujardin and Stone (1981) and Li (1986), which involves calculating the ratio of changes in pressure and flow during early systole, when the effect of wave reflection is minimal.

Like *Z*_c_, wave speed (*c*) may be considered an intrinsic property of a vessel, being dependent on vessel stiffness (i.e., elastic modulus), wall thickness and diameter, as expressed with the Moens–Korteweg equation (Mirsky, 1973). Wave speed differs subtly from pulse wave velocity (PWV), as routinely measured, in two respects. First, PWV is calculated as the time it takes for a wave to propagate from one location to another, whereas wave speed is a local quantity that is defined at every point along a vessel (similar to diameter). Second, the propagation speed of a wave is actually equal to *U* + *c* for a forward wave and *U* − *c* for a backward wave, where *U* is blood velocity (Parker, 2009). Hence, in principle PWV is determined by vessel properties (via *c*) and haemodynamics (via *U*), although *U* is generally much smaller than *c* (∼0.5 m/s vs. ∼5 m/s).

Various methods exist to estimate PWV and *c*, which were recently reviewed by Segers et al. (2020). We here focus on three approaches for estimating local *c*, which are most suited to wave separation analysis: (1) the ‘loop methods’ (e.g., PU loop); (2) the minimum energy or ‘sum of squares’ method, and (3) pressure-diameter or distensibility-based methods.

**FIGURE 5:**
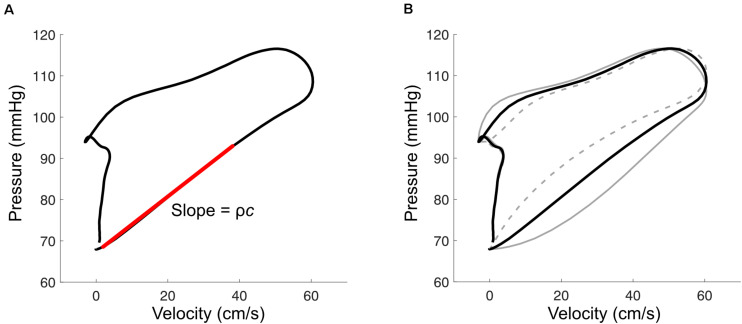
**(A)** With the pressure-velocity (PU) loop method, wave speed (*c*) is calculated from the slope of the early-systolic PU relation (red line), where ρ is blood density. **(B)** Misalignment of pressure and velocity signals introduces curvature in the early systolic relation. This example shows the effect of velocity leading or lagging pressure by 10 ms (solid and dashed gray lines, respectively).

The PU loop method is similar to the time domain approach for calculating *Z*_c_ mentioned above, and involves plotting pressure vs. velocity as shown in Figure 5A (Khir et al., 2001b). During early systole, the PU relation is relatively linear, which is presumed to be due to unidirectional wave travel, and the relation departs from this linear trajectory when reflected waves arrive at the measurement site. The most linear section is therefore selected and its slope (which is equal to ρ*c* if only forward waves are present) is divided by blood density (ρ) to obtain wave speed. Analogous methods have been described for diameter and velocity, the ln(D)U loop method (Feng and Khir, 2010), and for flow and area, the QA method (Rabben et al., 2004).

The accuracy of these ‘loop’ methods depends on three factors that should be carefully considered when applying them. First, signals must be time-aligned to correct for hardware-related lags or to combine signals that were acquired sequentially. Misalignment introduces curvature into the early-systolic PU relation (Figure 5B); one way to correct for these lags is to shift velocity with respect to pressure until the most linear relation is obtained (Khir et al., 2001b; Swalen and Khir, 2009). Second, the accuracy of these methods relies on the adequacy of the assumption that no reflected waves are present during early systole. Segers’ group has shown that the presence of reflected waves tends to cause over- and under-estimation of wave speed for the PU loop and QA loop methods, respectively, and importantly that reflections may be present even when the early systolic relation is linear (Swillens et al., 2013; Segers et al., 2014). Reflected waves in early systole may arise from proximity of the measurement site to reflection sites, such as the carotid bifurcation, or due to persistent effects of reflected waves from the previous cardiac cycle (i.e., the diastolic pressure decay); viscoelasticity and the pressure-dependence of wave speed may also have confounding effects (Alastruey, 2011; Mynard et al., 2011). Potential consequences of these phenomena include (1) incorrect alignment of signals due to real curvilinearity in the early systolic PU relation, (2) inaccurate wave speed estimation, and (3) wave separation that incorrectly suggests an absence of early reflected waves.

The requirement for unidirectional wave travel in the PU loop method was identified by Davies et al. (2006b) as a major barrier to wave speed estimation in coronary arteries, where there is no period of the cardiac cycle where this assumption can be confidently applied. These investigators therefore proposed the minimum net energy method (also referred to as the ‘sum-of-squares’ or ‘single-point’ method) in which wave speed is calculated as

(4) $$c=\frac{1}{\rho }\sqrt{\frac{\sum dP^{2}}{\sum dU^{2}}}$$

where *dP* and *dU* are incremental changes in pressure and velocity, with their squares summed over the cardiac cycle. This approach, along with a flow-area equivalent described by Quail et al. (2015), does not require unidirectional wave travel during a particular phase of the cardiac cycle, but instead minimizes the net energy of decomposed forward and backward waves throughout the whole cardiac cycle. This was based on a general observation that an incorrect wave speed tends to introduce self-canceling forward and backward waves (Siebes et al., 2009). Since self-canceling waves are minimized by the sum-of-squares method, its accuracy becomes limited when measurements are performed in close proximity to a reflection site, where self-canceling waves are expected (Borlotti et al., 2014). Although originally intended for use in coronary arteries, concerns have been raised about the accuracy of the sum-of-squares method in this setting during hyperemia, in the vicinity of a stenosis, and generally due to a preponderance of overlapping forward and backward waves (Kolyva et al., 2008; Rolandi et al., 2014). Hence, considerable challenges remain in obtaining a reliable single-point wave speed estimation technique for coronary arteries.

Distensibility-based methods for estimating wave speed have two major advantages over the ‘loop’ methods and sum-of-squares method in that (1) they require no assumptions about waves and (2) they do not require an accurate (mean) velocity signal, which can be difficult to acquire, as discussed above (Kowalski et al., 2017). Wave speed is related to distensibility (Δ) via the Bramwell–Hill equation (Bramwell and Hill, 1922),

(5) $$c^{2}=\frac{1}{\rho \Delta }=\frac{A}{\rho }\frac{dP}{dA}=\frac{1}{2\rho }\frac{dP}{dD}$$

where the last two expressions show that this method requires measurement of cross-sectional area (*A*) or diameter (*D*), in contrast to a velocity measurement required for the other techniques discussed above. Various implementations of Equation (5) exist, and these essentially differ in the segments of *P* and *D* used for calculating the derivatives; these include the *D*^2^*P* method that uses diastolic segments (Alastruey, 2011), the ln(D)P loop method that uses early systolic segments (Kowalski et al., 2017), and the distensibility coefficient method that uses the whole cardiac cycle (Segers et al., 2014). It could be argued that a disadvantage of these methods is that local pressure *and* diameter signals need to be measured, in addition to velocity or flow waveforms required for wave intensity and wave separation analyses. However, Kowalski et al. (2017) showed that a feasible approach is to estimate local (e.g., aortic, carotid, or femoral) pressure non-invasively by calibrating the local diameter (or area) waveform to mean and diastolic brachial blood pressures obtained with a standard cuff measurement. The benefit of this approach is not only that a more reliable wave speed is obtained (via a distensibility-based method), but that this wave speed can then be used to correct scaling errors in measured velocity or flow (Segers et al., 2014; Kowalski et al., 2017), which in turn may improve the accuracy of derived quantities such as wave intensity.

### Pressure-Only Wave Separation

Although a flow or velocity waveform is mathematically required to perform wave separation, Westerhof et al. (2006) introduced the concept of replacing measured flow with a synthesized waveform because the flow waveform displays relatively little variation between individuals. With this approach, wave analysis can be conducted with only a pressure waveform and wave speed or characteristic impedance are not required. Westerhof et al. (2006) originally proposed using a triangle to approximate systolic flow, with a base spanning from the start of the pressure upstroke to the dicrotic notch, and a peak at 30% of ejection time or at the point of pressure inflection. Kips et al. (2009) subsequently found that a population-average flow waveform resulted in better accuracy for estimating reflection magnitude, but found poor correlation between actual and estimated reflection transit time for both triangular and average flow waveforms. Parragh et al. (2015) also cautioned against using a one-size-fits-all flow waveform in settings where ventricular outflow patterns may be abnormal, such as heart failure with reduced ejection fraction. They instead used a personalized approximation of the flow waveform, generated with a modified windkessel (ARCSolver) model (Hametner et al., 2013). While these pressure-only approaches should be used with caution due to the assumptions required, the benefit of broad applicability has been demonstrated in numerous large cohort studies where flow signals are unavailable (Wang et al., 2010; Chirinos et al., 2012; Weber et al., 2012; Zamani et al., 2014; Hametner et al., 2015; Sluyter et al., 2017).

## Wave Intensity

Wave intensity analysis (WIA) was introduced by Parker et al. (1988) as an intuitive time domain method for visualizing and quantifying arterial waves. WIA is based on the one-dimensional equations of flow and uses the method of characteristics (familiar to the fluid dynamics community) to reveal forward- and backward-propagating ‘information’ that is not obviously apparent when looking at raw pressure and velocity signals (Parker and Jones, 1990; Parker, 2009). Wave intensity (*dI*) is simply defined as

**FIGURE 6:**
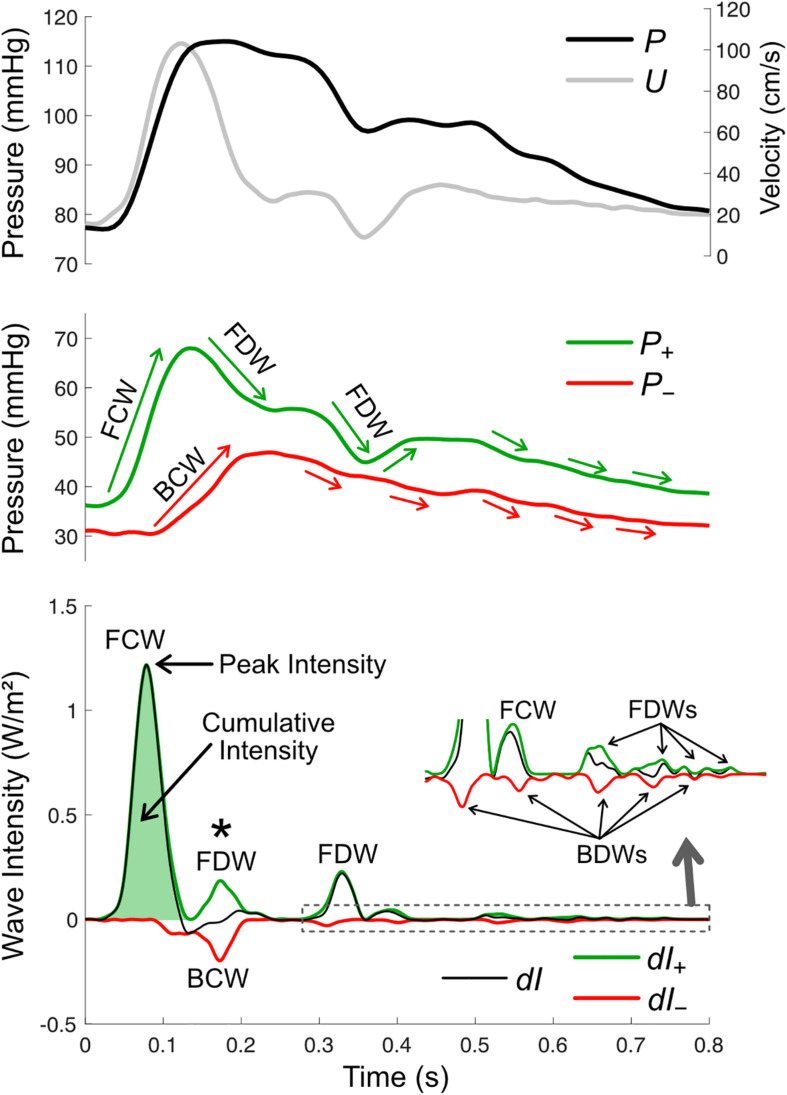
Wave intensity analysis in the carotid artery, based on pressure (*P*, derived from measured diameter), mean velocity (*U*), forward and backward components of pressure (*P*_+_ and *P*_–_), net wave intensity (*dI*) and forward and backward wave intensity components (*dI*_+_ and *dI*_–_). There are four possible wave types, forward compression waves (FCW), forward decompression wave (FDW), backward compression wave (BCW), and backward decompression wave (BDW). Wave size is quantified via peak or cumulative intensity. Whether a wave is a compression or decompression wave can be judged by its effect on the respective pressure components (green and red arrows in the middle panel; note that waves shown in the zoomed inset in the bottom panel are not labeled in the middle panel). The asterisk (*) indicates a time where a BCW and FDW arrive at the measurement site around the same time and therefore wave separation is essential for revealing the presence/magnitude of these waves.

(6) dI=dPdU

where *dP* and *dU* are incremental changes in pressure and velocity, respectively, over a sample interval. An example of wave intensity in the carotid artery is shown in Figure 6. The distinct peaks are referred to as ‘waves,’ and these arise from the cumulative effect of many ‘wavelets’ or infinitesimal ‘wavefronts’ that are each associated with a small change in pressure (*dP*) and velocity (*dU*) that propagates along the vessel. As a crude analogy, a wave may be compared with a staircase while a wavefront is a single step. The value of wave intensity at a given time may be compared to the height of a particular step, while ‘cumulative intensity’ (the integrated area under a wave) is analogous to the overall height of the staircase. The units of wave intensity (Watts/m^2^ or Joules per second per square meter) indicate the rate at which wave energy passes through a given cross-section. These are the same units as sound intensity, which determines the ‘loudness’ of a sound wave, or radiant flux which determines how much light energy falls onto a solar panel. Importantly, however, wave intensity refers to the energy flux of a propagating wave, not the energy of flowing blood.

Although WIA is assessed at a single site, the direction in which a wave propagates is easily discernable, as a forward wave is always positive and a backward wave is always negative. What is not clear from the wave intensity signal alone is whether a wave has a pressure-increasing effect (called a *compression wave*) or a pressure-decreasing effect (a *decompression wave*, at times also referred to as an ‘expansion’ or ‘suction’ wave). The wave type must therefore be determined via wave separation of the pressure waveform. Then, for example, a forward wave is identified as a compression wave if it coincides with an increase in *P*_+_, while a backward wave is identified as a decompression wave if it coincides with a decrease in *P*_–_ and so on (Figure 6).

One drawback of the original definition of wave intensity is that the numerical value of *dPdU* depends on the sampling frequency of the measured signals, which is extraneous to the underlying physiological quantity. In our stair analogy, it would mean that the height of the staircase is dependent on the step size, which is chosen arbitrarily. For this reason, Ramsey and Sugawara (1997) introduced a time-corrected form of wave intensity (designated *wi* instead of *dI*) that employs the time derivatives of pressure and velocity, i.e.,

(7) $$wi=\frac{dP}{dt}\frac{dU}{dt}$$

This signal is simply a scaled version of *dI* but is independent of sample time (*dt*), being analogous to using the slope of the staircase rather than the height of individual steps (the total height now being independent of step size). Khir and Parker (2005) pointed out that, unlike *dI*, which has units representing power flux, the units of *wi* (W/m^2^/s^2^) lack such a physical meaning. The sample rate dependence of *dI* and the unclear physical meaning of *wi* units thus creates a theoretical trade-off that is presently an unresolved issue in the WIA field; however, the issue has no bearing on the analysis or interpretation of wave dynamics in practice.

As for pressure and flow, *net* wave intensity (*dI*) may also be subjected to wave separation, providing distinct forward and backward components (*dI*_+_ and *dI*_–_, respectively). These components are calculated via

(8) $$dI_{\pm }=\pm \frac{1}{4\rho c}{\left(dP\pm \rho cdU\right)}^{2}$$

where, similar to pressure and flow-velocity separation, an estimate of wave speed (*c*) is required (Parker, 2009). While net wave intensity indicates whether forward or backward waves are dominant at a particular time, wave separation further allows quantification of potentially overlapping or partially overlapping forward and backward waves. Wave separation may therefore have a substantial impact on calculated wave reflection indices or other wave ratios (an example of this is indicated with an asterisk in Figure 6).

### Non-invasive Wave Intensity

The ability to measure wave intensity non-invasively is pivotal to clinical translation. This can be achieved by measuring pressure and velocity waveforms sequentially via applanation tonometry and Doppler ultrasound, then aligning these signals before calculating wave intensity (Zambanini et al., 2005). However, a more common approach, described in the first non-invasive WIA study by Niki et al. (1999), is to employ a combined echo-tracking and Doppler system to obtain carotid arterial diameter and velocity waveforms simultaneously, with the pressure waveform estimated by calibrating the diameter waveform to systolic and diastolic pressure from an arm cuff. This technique was developed and incorporated into a commercial ultrasound scanner and has been used in numerous clinical WIA studies of the carotid artery (Niki et al., 2002, 2005, 2017; Babcock et al., 2014; Tanaka et al., 2014; Nogami et al., 2018; Chiesa et al., 2019). This approach assumes that pressure and diameter waveforms are identical, and the calibration method does not account for the difference between brachial and carotid systolic pressures caused by pulse amplification, which exhibits individual variability (Segers et al., 2009). Several techniques have also been proposed to estimate central wave intensity non-invasively from peripheral measurements, with central aortic or coronary blood pressure being estimated with a generalized transfer function or supra-systolic oscillometry, and aortic flow via the reservoir-excess pressure approach (Broyd et al., 2016; Smolich and Mynard, 2016; Hughes et al., 2020); given the relatively high sensitivity of wave intensity to measurement errors, these techniques require further validation before widespread adoption.

In additional efforts to foster clinical translation, a number of wave intensity surrogates have been described that avoid issues related to pressure calibration. Feng and Khir (2010) described a version of wave intensity suited to ultrasound studies, in which vessel diameter is used in place of pressure. Similarly, Biglino et al. (2012) used the natural logarithm of area instead of pressure, which is suited to phase contrast MRI. These alternative definitions of wave intensity and associated equations for performing wave separation are summarized in Table 1. While these methods can be derived from the one-dimensional flow equations, a limitation is that the units of diameter- or area-based ‘wave intensity’ (m^2^/s and m/s, respectively) are not those of intensity (W/m^2^), and hence their physical interpretation is uncertain.

### Wave Power

Wave power is an alternative to wave intensity that uses pressure and volumetric flow signals (*d*π = *dPdQ*) and has a number of advantages (Mynard and Smolich, 2016). First, as the name suggests, wave power has the physically meaningful units of power (Watts) and is therefore not sensitive to cross-sectional area variations (e.g., within vessels or between individuals). Second, in experimental studies, measured flow can be used directly for wave analysis without requiring derivation of velocity, which often requires error-prone assumptions about cross-sectional area (Kowalski et al., 2019b). Third, wave power is conserved at junctions and therefore the distribution of wave power to different vessel branches can be quantified. For example, unlike wave intensity which is not conserved, it has been possible to meaningfully quantify the percentage of ascending aortic wave power that passes into the carotid artery and how this changes with pharmacological intervention (Londono-Hoyos et al., 2018). Finally, wave power is linked to the hydraulic pressure power of flowing blood (Π_P_ = *PQ*), which determines total ventricular workload (Laskey et al., 1985). While hydraulic power has traditionally been decomposed into steady and oscillatory components (Milnor et al., 1966; Burattini and Campbell, 1999), Mynard and Smolich (2016) showed that power can also be separated into forward and backward wave components (Π_P__±_, see Table 1). Moreover, an incremental change in Π_P__±_ is proportional to wave power divided by the associated fractional change in pressure or flow components.

## Interpretation of Wave Patterns

Having reviewed the various approaches to quantifying waves, this section provides an overview of the biomechanical mechanisms that generate waves and factors that affect wave magnitude.

### How Are Waves Generated?

There are four recognized mechanisms that generate waves in blood vessels: (1) wave generation by a pump, (2) inertial effects, (3) wave potential gradients and (4) wave reflection.

#### Wave Generation by a Pump

**FIGURE 7:**
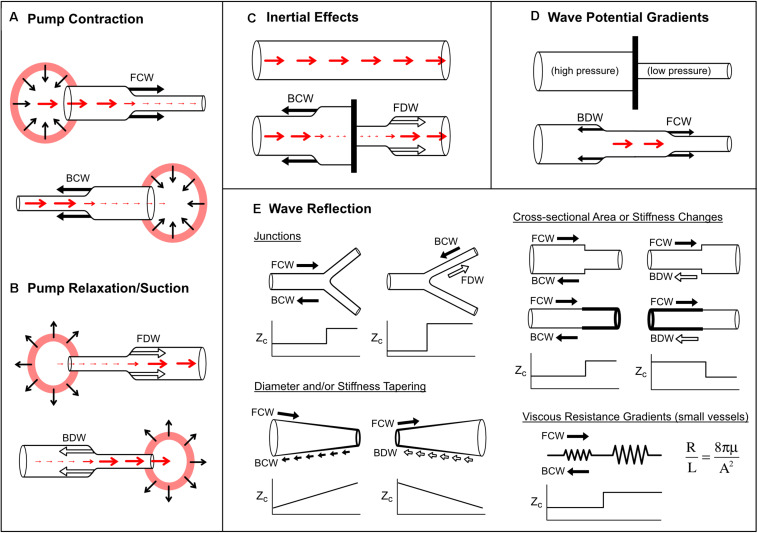
Mechanisms that produce pressure/flow waves in blood vessels. See text for detailed explanation. Red arrows indicate blood flow or velocity (larger arrow means higher flow/velocity). Solid black arrows indicate compression (pressure-increasing) waves, while outlined black arrows indicate decompression (pressure-decreasing) waves. Panels **(A,B)** illustrate wave generation via active pump contraction and relaxation respectively. In panel **(C)**, a barrier is placed in a vessel that contains flowing blood. In panel **(D)**, a barrier is removed between two vessels containing blood at different pressures. In panel **(E)**, increases or decreases in characteristic impedance (*Z*_c_; or combined *Z*_c_ in the case of the bifurcation) are plotted versus horizontal position. Viscous resistance per unit length (R/L, given by Poiseuille’s law, where μ is blood viscosity and A is cross-sectional area) is small in large vessels but becomes dominant in small vessels.

Active contraction of a pump causes a pressure increase and therefore a compression wave (Figure 7A); however, whether a flow-increasing forward compression wave (FCW) or flow-decelerating backward compression wave (BCW) is produced depends on which direction the pump is ‘facing’ with respect to the direction of blood flow. For example, commencement of left ventricle (LV) ejection causes an increase in aortic pressure and flow that begins at the aortic valve and propagates away from the heart, giving rise to a FCW (Parker et al., 1988; Jones et al., 2002; Penny et al., 2008). A similar wave is observed in pulmonary arteries due to right ventricular contraction (Hollander et al., 2001; Su et al., 2016). On the other hand, although atrial contraction aids ventricular filling, the absence of an atrial inflow valve means that this contraction also generates a BCW that propagates into the systemic/pulmonary veins in a direction that is opposite to mean blood flow (Hellevik et al., 1999; Smiseth et al., 1999; Hobson et al., 2007; Mynard, 2011). Other pump phenomena that generate BCWs in arteries include the active compression of the coronary microvasculature by contracting myocardium (Davies et al., 2006a; Mynard et al., 2018) and the inflation of an intra-aortic or para-aortic balloon pump (Kolyva et al., 2009; Lu et al., 2012).

After a pump has contracted, relaxation causes pressure to fall and therefore generates a decompression wave (sometimes called a ‘suction wave’) (Figure 7B). Decompression waves are generated by atrial relaxation (Smiseth et al., 1999; Mynard, 2011), release of extravascular pressure on intramyocardial vessels by the relaxing ventricle (Davies et al., 2006a; Mynard et al., 2018) and deflation of aortic balloon pumps (Kolyva et al., 2009; Lu et al., 2012). In the first paper utilizing wave intensity analysis, entitled “What stops the flow of blood from the heart?”, Parker et al. (1988) showed that a forward decompression wave (FDW) is primarily responsible for the deceleration of systolic flow in arteries, not a reflected wave as had been widely believed. A similar FDW was also later reported in pulmonary arteries (Hollander et al., 2001; Su et al., 2016). These FDWs precede and appear to cause valve closure, and are often thought to arise from the onset of ventricular relaxation, which involves elastic recoil and myocardial untwisting (Parker and Jones, 1990; Nakayama et al., 2005; Sun et al., 2006; Parker, 2009; Babcock et al., 2014). However, the extent to which the FDW is caused by relaxation versus inertial effects is somewhat controversial, as will be discussed in the next section.

A quantitative dependence of arterial FCW and FDW on ventricular pump function was first evidenced in humans by Ohte et al. (2003), who found that the aortic FCW was correlated with maximal LV dP/dt (*r* = 0.74) and aortic FDW was negatively correlated with the time constant of LV relaxation (τ, *r* = −0.77). However, Nakayama et al. (2005) subsequently found a much weaker correlation (*r* = 0.46) between FCW and LV end-systolic elastance (Ees), a relatively load-independent index of contractility, and no significant correlation between FDW and τ. Adjusting for end-diastolic volume improved correlation coefficients substantially, to ∼0.9 for FCW vs. Ees and −0.7 for FDW vs. τ, suggesting that these waves are dependent on preload as well as ventricular pump function (inotropy and lusitropy). Others have shown that an acute increase in ventricular afterload (via aortic constriction) decreases FCW amplitude (Khir and Parker, 2005; Mynard et al., 2017). Conflicting data exists on whether an afterload increase causes an increase (Mynard et al., 2017) or decrease (Khir and Parker, 2005) in FDW amplitude, which may suggest a complex dependence of FDW on afterload that requires further study. Despite its load dependence, FCW may have potential as a sensitive and early marker of ventricular systolic dysfunction, with myocardial apoptosis shown to correlate with carotid FCW (*r* = −0.69) but not ejection fraction (a commonly used clinical index of systolic function) in rabbits (Zhang et al., 2014). In humans, the FCW and FDW both decrease with advancing age, but it is presently unclear whether this is due to changes in pump function, loading conditions (e.g., increasing aortic stiffness), or both (Bhuva et al., 2019; Li et al., 2019).

#### Inertial Effects

Consider a ‘thought experiment’ in which a constant forward flow is initially present in a long tube (Figure 7C). If a barrier is suddenly inserted in the middle of the tube, then clearly the flow must stop. Flow-decreasing waves must therefore be generated on both sides of the barrier, namely a decompression wave in the forward direction (FDW) and a compression wave in the backward direction (BCW). These waves arise from inertial effects. When the barrier is put in place, the fluid ‘wants’ to keep flowing under its own momentum, but accumulation of the fluid behind the barrier leads to a pressure build up (and hence a BCW), while inertial flow continuing past the barrier causes a suction effect, causing pressure to fall (leading to the FDW).

Sugawara et al. (1997) proposed that the late-systolic FDW in the aorta may arise primarily from inertial effects rather a loss of the tension bearing ability of the myocardium (i.e., relaxation). A helpful way to understand the generation of waves by the ventricle is to consider the degree of matching between myocardial contraction rate and outflow. During early systole, the rate of myocardial contraction exceeds outflow (which is initially zero) and therefore causes acceleration and imparts momentum to blood, generating the FCW. Then, in mid-systole, there is a virtual absence of forward waves, suggesting that the rate of myocardial contraction ‘matches’ outflow, hence forward momentum is maintained. However, later in systole, slowing contraction cannot match outflow, and therefore forward momentum cannot be maintained. Similar to the aforementioned example of a tube with flow halted by insertion of a barrier, outflow in the ventricle cannot continue indefinitely and therefore pressure and flow must fall, thus an FDW is generated. That inertial effects are primarily responsible for the FDW is suggested by data from multiple sources indicating that the aortic FDW starts 70–164 ms before aortic valve closure, whereas the onset of LV relaxation (quantified by the time of peak torsion or myocardial shortening) occurs up to ∼30 ms before, to 80 ms *after*, aortic valve closure (Table 2).

**TABLE 2:** Timing of aortic forward decompression wave duration and onset of LV relaxation.

Aortic FDW duration	References
72 ms (human)	Figure 3 in Davies et al. (2012)
122 ms (human)	Figure 1 in Hametner et al. (2017)
164 ms (human)	Figure 2 in Koh et al. (1998)
97 ms (human)	Figure 5 in Khir et al. (2001a)
36–66 ms (dog)	Figures 4, 5 in Khir and Parker (2005)
37 ms (sheep)	Figure 3 in Mynard and Smolich (2016)
**Time from peak LV torsion to aortic valve closure**	
14 ms (human)	Table 1 in Notomi et al. (2006a)
0–7 ms (human)	Figure 1 in Notomi et al. (2006b)
27 ms	Dong et al. (2001)
**Time from peak myocardial shortening to aortic valve closure**	
25 to −80 ms (human)	Figure 4D in Zwanenburg et al. (2004)
27 to −21 ms (human)	Figure 6 in Mada et al. (2015)

*FDW, forward decompression wave; LV, left ventricle. A negative time indicates peak LV torsion or shortening occurs after aortic valve closure. Note that it is generally thought, and modeling studies suggest (Mynard and Smolich, 2015), that the end of the aortic FDW corresponds closely to the time of valve closure.*

A striking example of inertial effects producing a FDW in the aorta was provided by Penny et al. (2008). Infusion of incremental doses of dobutamine in sheep caused increases in the force of LV contraction and exponential rises in initial FCW. However, the substantial momentum imparted to blood during this early phase could not be maintained throughout systole, leading to an abrupt fall in pressure and flow (and hence a FDW) during mid-systole. It was originally speculated that this mid-systolic FDW arose from inertial effects, whereas the late-systolic FDW arose from LV relaxation. However, based on the data in Table 2, it is possible that both waves were produced by inertial effects.

Finally, we note that compression waves also arise from inertial effects when flow approaches a barrier or closed chamber (Figure 7C). For example, modeling studies suggest that this mechanism may play a key role in decelerating blood entering the atria (Mynard, 2011).

#### Wave Potential

Wave potential is a recently coined term referring to the absolute values of *P*_±_ and *Q*_±_, as discussed in more detail in a later section. Mynard and Smolich (2014b) showed that any spatial difference (gradient) of wave potential generates waves and that the pressure and flow effects of these waves can be predicted. For example, Figure 7D shows a situation where a vessel is completely obstructed (e.g., with a clamp), preventing flow (*Q*_1_ = *Q*_2_ = 0) despite the presence of a pressure difference (*P*_1_ > *P*_2_). Due to this pressure difference, the equations in Table 1 show that the values of *P*_±_ and *Q*_±_ on either side of the barrier differ. When the barrier is removed, the spatial differences in pressure and wave potential (which can no longer be sustained) are eliminated by the generation of forward compression and backward decompression waves.

#### Wave Reflection

Wave reflection occurs when a propagating wave encounters a change in characteristic impedance (*Z*_c_) or its inverse, characteristic admittance (*Y* = 1/*Z*_c_). Recalling that *Z*_c_ in large vessels is proportional to wave speed and inversely proportional to cross-sectional area, changes in the stiffness or caliber of a vessel may produce a reflection-producing ‘impedance mismatch’ (Figure 7E). The extent of the mismatch determines the degree to which a wave is reflected, quantified via a *pressure reflection coefficient* (*R*_P_),

(9) $$R_{\text{P}}=\frac{Y_{0}-Y_{1}}{Y_{0}+Y_{1}}$$

for a wave that is propagating from admittance *Y*_0_ to *Y*_1_. An extreme example is a complete blockage or occlusion, which presents an infinite impedance or zero admittance (i.e., *Y*_1_ = 0); hence *R*_P_ = 1. This means that, for an incident wave causing a pressure rise of 10 mmHg, the reflected wave will produce an additional 10 mmHg pressure rise. The opposite extreme is where a wave encounters an infinite opening, such as a tube that is cut and open to the atmosphere. In this case *Y*_1_ → ∞ and hence *R*_P_ = −1; hence the pressure change associated with the incident wave will be entirely negated by the pressure change associated with the reflected wave. Other reflection coefficients can be defined for flow (*R*_Q_ = −*R*_P_), wave intensity and hydraulic power (*R*_wi_ = *R*_Π_ = *R*_Q_*R*_P_), but pressure coefficients are used most frequently.

In blood vessels, impedance mismatching may take various forms. Branch junctions may cause an impedance mismatch if the combined admittance of the daughter vessels does not match the admittance of the parent vessel (Figure 7E). Importantly, arterial junctions are typically well-matched in the forward direction but not in the backward direction. This has a clear teleological benefit for ventricular afterload, since reflected waves become ‘trapped’ downstream and find it ‘difficult’ to return to the heart (Khir and Parker, 2005; Davies et al., 2012).

Vessel tapering produces an impedance mismatch that is distributed over its length (Figure 7E). In this case, wave reflection does not occur at a discrete point, but many small reflections are produced in a continuous manner. Interestingly, Segers and Verdonck (2000) showed that distributed reflection due to tapering may have similar effects on measured pressure/flow as a discrete reflection, and hence whether wave reflection is discrete or distributed may not be discernable from measurements at a single site. The extent to which tapering contributes to arterial wave reflection is uncertain. Although many arteries are tapered, this may in fact be an impedance-preserving feature due to the presence of side branches. Indirect evidence for this is the gradual decrease in mean blood velocity in progressively distal arterial locations (Milnor, 1990), whereas if side branches were few or absent in a tapered vessel, velocity would be expected to increase due to convective acceleration.

A stiff vascular segment may occur in certain disease conditions or after surgery due to formation of scar tissue (e.g., at the site of an aortic coarctation repair), while a stent similarly represents a stiff segment compared with the surrounding vessel. Although some reflection is expected in these settings, it is important to note that a stent creates two sites of impedance mismatch, the stiffness increase at the proximal end (vessel-to-stent) and the stiffness decrease at the distal end (stent-to-vessel). The reflection coefficients at these interfaces are therefore positive and negative, respectively, and therefore reflected waves from the two sites tend to cancel out if there is a negligible delay between them. Short stiff segments may therefore produce only minor wave reflection effects overall (Taelman et al., 2015). A pathological narrowing (stenosis) or expansion (aneurysm) of a vessel present regions of high and low impedance, respectively, and may lead to wave reflection (Stergiopulos et al., 1996; Swillens et al., 2008; Sazonov et al., 2017).

#### Viscous Resistance in Arterioles

Characteristic impedance in a vascular segment with non-leaky walls is determined by three factors, namely the inertia of blood (*L*), the compliance of the vessel (*C*), and the resistance to flow caused by blood viscosity (*R*), as follows,

(10) $$Z_{\text{c}}=\sqrt{\frac{R+j\omega L}{j\omega C}}$$

where ω is angular frequency and $j=\sqrt{-1}$ (Pollack et al., 1968). In large arteries such as the aorta, resistance is negligible, and therefore Equation (10) reduces to $Z_{\text{c}}=\sqrt{L/C}$. Noting that *L* = ρ*l/A* and *C* = *Al/*(ρ*c*^2^), where ρ is blood density, *l* is length, *A* is cross-sectional area, and *c* is wave speed, it can be shown that *Z*_c_ = ρ*c/A* as mentioned previously. However, because resistance is inversely proportional to the fourth power of radius (Poiseuille’s law), viscous resistance becomes the main determinant of characteristic impedance in small vessels.

Resistance-related increases in *Z*_c_ may therefore produce wave reflection. Indeed, Nichols and O’Rourke (2011) stated that “high-resistance arterioles are considered to be the major sites of wave reflection in the circulation.” This was based on the precipitous fall in pressure in these vessels and, more directly, the finding that vasoconstrictors and vasodilators that act mainly on small muscular arteries and arterioles cause a major increase and decrease in wave reflection, respectively (O’Rourke and Taylor, 1966; van den Bos et al., 1982).

### Factors Influencing Wave Magnitude

When performing wave intensity or wave power analysis, one might expect that the magnitude of a measured wave will be directly determined by the magnitude of the force that generated it, such as the strength of contraction or rate of relaxation of a pump (including load-dependence), the magnitude of the inertial force, or the degree of impedance mismatch. Although these may be the dominant factors governing wave magnitude in many settings, there are a number of other phenomena that can have a substantial influence on wave magnitude.

#### Wave Amplification and Attenuation Due to a Non-linear Pressure-Area Relation

**FIGURE 8:**
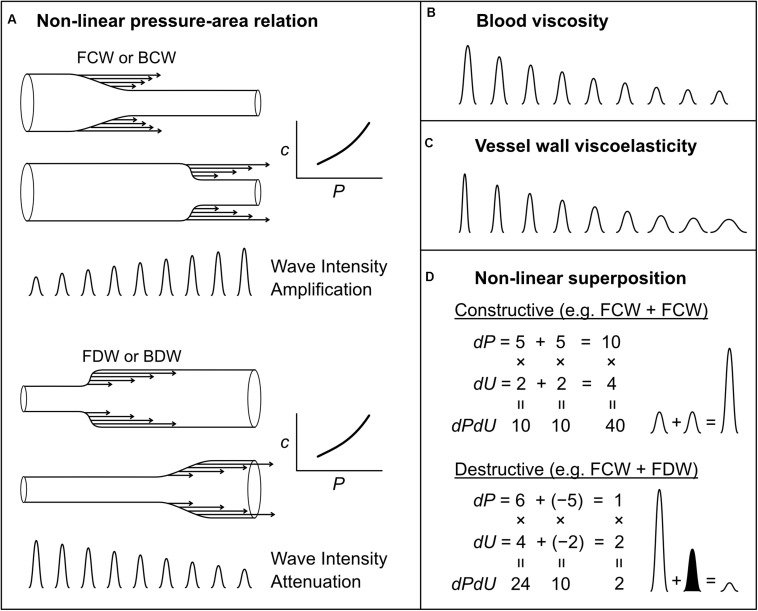
Mechanisms that affect wave intensity magnitude as a wave propagates **(A–C)** or when two waves propagating in the same direction, but generated from separate mechanisms, combine constructively or destructively **(D)**. Waves shown in **(B,C)** could be any wave type (FCW, FDW, BCW, or BDW). In the examples of wave superposition shown in **(D)**, the shaded and unshaded waves indicate an FDW and FCW, respectively.

Mynard et al. (2008) showed that waves can grow (amplify) or shrink (attenuate) as they propagate in a vessel with a non-linear pressure-area relation. Although this relation may be approximately linear over a limited range, all vessels have non-linear pressure-area relations because at low pressures they become highly compliant and collapse, while at high pressures there is a progressive shift in load-bearing from elastin to the stiffer collagen fibers. The slope of the pressure-area relation determines both the area compliance (*C* = *dA/dP*) and wave speed [*c*^2^ = *A*/(ρ*C*)] of a vessel, and hence any non-linearity in this relation means that wave speed is pressure-dependent. Consider a compression wave (FCW or BCW) that causes an increase in pressure by 4 mmHg overall, composed of 4 individual wavelets that each contribute 1 mmHg (Figure 8A; for illustration purposes, we here relax the principle that wavelets are infinitesimally small). The last wavelet (i.e., which contributes the last 1 mmHg rise) will propagate at a faster speed than the first wavelet, because the wavelets that came before it increased pressure and hence instantaneous wave speed. As a consequence, the overall rate at which pressure changes (*dP/dt*) increases as the wave propagates. Since wave intensity is equal to *dP/dt* × *dU/dt*, this phenomenon causes the intensity of a compression wave to increase (or amplify) as it propagates. The opposite is true for decompression waves, for which the last wavelet propagates more slowly (since it exists at a lower pressure) than that of the first wavelet; hence, the pressure slope and wave intensity decrease as the wave propagates (Figure 8A).

This amplification of compression waves and attenuation of decompression waves has important implications for how one assesses wave reflection (Mynard et al., 2008). For example, if a FCW propagates along a vessel and is reflected as a BCW that propagates back to the measurement site, the ratio of BCW/FCW wave intensities will not be a reliable index of the reflection if the waves have been amplified during propagation (unless the amount of amplification is known). This issue is not purely academic, but is a likely explanation for the curious finding of BCW/FCW ratios significantly greater than 1 in pulmonary arteries of near-term fetal lambs (Smolich et al., 2008, 2009). Importantly, Mynard et al. (2008) showed that non-linear effects do not affect the overall pressure effect of a wave, only the rate at which pressure changes. We therefore recommend that wave reflection be quantified from the ratio of the pressure effects of forward and backward waves rather than the wave intensity (peak or cumulative intensity) of those waves.

#### Wave Attenuation Due to Blood Viscosity and Vessel Wall Viscoelasticity

While the non-linear amplification/attenuation effect discussed above is energy conserving, with energy becoming more or less ‘concentrated’ in time (increasing or decreasing wave intensity), energy dissipation in the form of heat also occurs in blood vessels due to viscous friction in moving blood and in the stretching/relaxing vessel wall. Many studies have investigated the influence of these factors on wave attenuation, but will not be reviewed in detail here (Westerhof and Noordergraaf, 1970; Salotto et al., 1986; Horsten et al., 1989; Reuderink et al., 1989; Bertram et al., 1997; Alastruey et al., 2011, 2012; Wang et al., 2016). In brief, both sources of dissipation cause attenuation of compression and decompression waves, leading to an exponential fall in the magnitude of propagating waves; this effect is minor in large vessels, but has a substantial impact on small vessel haemodynamics (Salotto et al., 1986; Segers et al., 1995; Feng and Khir, 2007, 2008; Mynard et al., 2008). The effects of blood viscosity and wall viscosity are not identical; wall viscosity causes both attenuation (decreased magnitude) and dispersion (spreading out or widening) of waves, whereas blood viscosity predominantly causes only attenuation (Figures 8B,C) (Alastruey et al., 2012).

#### Non-linear Wave Superposition Effects

When interpreting wave patterns, one of the mechanisms responsible for generating waves mentioned above may be responsible for a given wave, but it is also possible that multiple mechanisms act together to produce a particular wave. This issue was recently identified in the context of coronary arterial wave intensity analysis, which is characterized by a large backward decompression wave (BDW) that is mainly responsible for the early diastolic surge in coronary flow (Mynard et al., 2018). It was previously thought that this BDW arose solely from an active suction effect in the intramyocardial circulation caused by myocardial relaxation (Davies et al., 2006a; Lee et al., 2016; Raphael et al., 2016). However, evidence from experimental and modeling data suggested that the coronary BDW actually arises from a combination of (1) the active relaxation effect and (2) simultaneous reflection of the aortic FDW that is transmitted into coronary arteries (Mynard et al., 2018).

More generally, it was demonstrated that when two mechanisms contribute to a particular wave, the resultant wave intensity is not the linear sum of the intensities of the waves that would be produced by each mechanism independently. For example, if two mechanisms both produce waves causing a pressure change (*dP*) of 5 and velocity change (*dU*) of 2 (ignoring units), then the wave intensity (*dPdU*) arising from each mechanism in isolation would be 5 × 2 = 10; however, the wave intensity resulting from the simultaneous action of both mechanisms, resulting in ‘constructive interference,’ would be (5 + 5) × (2 + 2) = 40, which is twice the linear sum of the intensities, i.e., 10 + 10 = 20 (Figure 8D). Similarly, two processes that have opposing pressure effects will have a non-linear canceling effect (destructive interference), which can lead to ‘concealment’ of those processes in the wave patterns (Mynard et al., 2018) (Figure 8D). In the coronary arteries, it may be possible to ‘disentangle’ multiple mechanisms underlying observed waves (Mynard et al., 2018). However, whether the non-linear superposition of two or more wave-generating mechanisms is relevant in other settings, and the extent to which those mechanisms could be disentangled, is a potential avenue for future research.

#### Wave Frequency

One property of wave intensity is that wave magnitude is profoundly affected by wave frequency, that is, the rapidity with which pressure and velocity change. One downside of this property is that it is possible for a wave with a large amplitude to have a very small overall effect on pressure and flow (small but rapid change), while conversely a large pressure/flow change may be associated with indiscernible wave intensity (large but slow change). A key example of the latter is the diastolic period, when *P*_±_ changes are substantial but relatively slow, which could lead to the impression that no waves are present during this time. However, close inspection reveals that *wi*_±_ is non-zero during this time (Mynard and Smolich, 2014b) (see also Figure 6). This issue is not resolved by calculating cumulative intensity (i.e., wave area) rather than peak intensity, and we therefore recommend always reporting the overall pressure or velocity ‘impact’ of waves (commonly designated Δ*P*_±_ or Δ*U*_±_) in addition to their intensity.

## Wave Potential

Mean or absolute values of blood pressure and flow have historically been neglected when analyzing pressure/flow waves in the arterial system. For example, absolute values have no impact on wave intensity, which is calculated from *changes* in pressure and flow-velocity. Similarly, when performing wave separation in the frequency or time domain, only the oscillatory component of pressure has been considered relevant (Westerhof et al., 1972; Li, 1986). However, there has been a growing recognition that, physically, waves must be linked with mean pressure, since the latter ultimately arises from pulsatile pressure/flow ‘inputs’ (wave generation) from the ventricle; when these inputs cease, pressure does not remain constant, but falls to a much lower level (Jellinek et al., 2000). Conversely, after a period of asystole, recommencement of wave generation by the ventricle restores mean pressure (Jellinek et al., 2000; Alastruey et al., 2009).

The link between waves and the absolute values of pressure and flow was explored by Mynard and Smolich (2014b), who proposed the concept of ‘wave potential.’ This provided a physical meaning to the absolute values of *P*_±_ and *Q*_±_ for the first time. In brief, just as pressure represents flow potential (i.e., flow is generated when a pressure difference exists between two locations), so the absolute values of *P*_±_ and *Q*_±_ represent the potential for pressure and flow wave generation at that time (arrows in Figure 4). Extending the staircase analogy discussed earlier, whereas waves correspond to stairs, wave potential represents the absolute height above ground level (the potential for how far one could fall!). Here, the ‘ground level’ is defined as the state in which no waves could be generated (one could not fall further); that is, when flow is zero and pressure equals ‘undisturbed pressure’ (*P*_ud_). The latter signifies the pressure that would exist if no pressure differentials existed throughout the circulation, and may therefore be identified with mean circulatory pressure (*P*_mc_) (Mynard and Smolich, 2014b). Alternatively, *P*_ud_ could be identified with the zero-flow or asymptotic pressure that is reached after a long period of asystole, which may not be equal to *P*_mc_ (Hughes and Parker, 2020).

Regardless of the pressure ‘ground’ that is chosen, it has been shown that wave potential closely relates to the stored pressure, blood volume, and hydraulic energy (i.e., potential for hydraulic work) in a distended arterial segment (Mynard and Smolich, 2014b, 2016) (Figure 7C). The concept therefore allows for a unified wave-based description of both wave phenomena and the windkessel effect, where the latter refers to the storage and discharge of blood in the elastic arterial reservoir. During systole, waves are responsible for filling the arterial reservoir because these increase pressure and to some extent become trapped in the arterial network due to multiple reflections and re-reflections, thus building up wave potential. Then during diastole, discharge of the arterial reservoir occurs due to leakage of wave potential, since waves are only partially reflected at the distal outlets of the network (Mynard and Smolich, 2014b, 2017).

## Waves and the Reservoir Pressure

**FIGURE 9:**
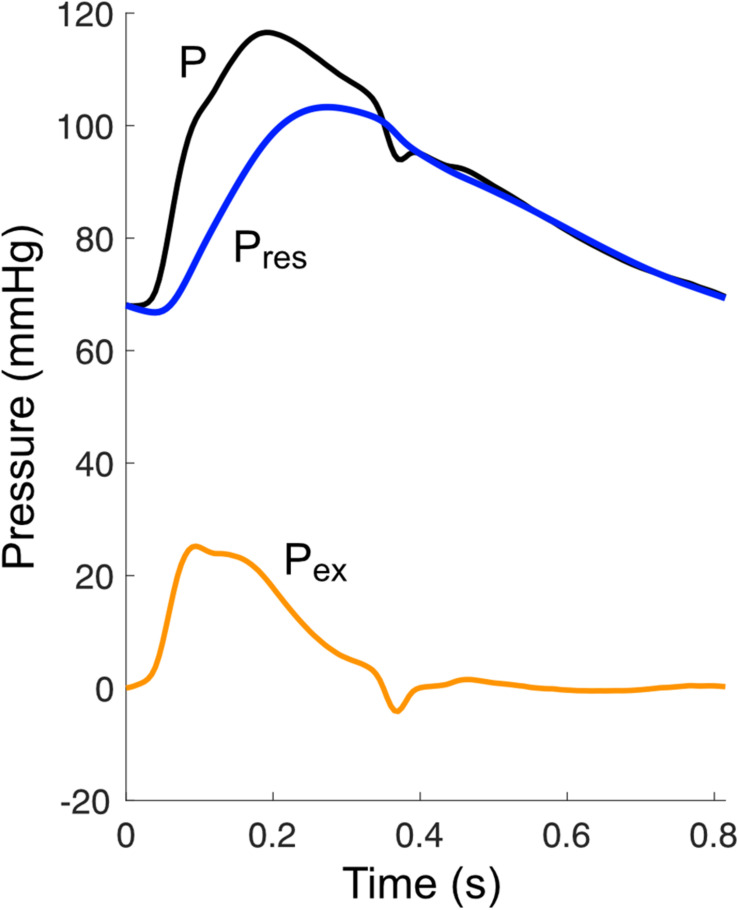
Decomposition of pressure (*P*) into a reservoir pressure (*P*_res_) and an excess pressure (*P*_ex_).

Another approach that has attempted to unify windkessel and wave models of haemodynamics is the reservoir-wave paradigm. Initially termed the windkessel-wave approach, Wang et al. (2003) separated measured pressure into two components, a ‘windkessel pressure’ calculated via a two-element windkessel model and considered to govern the windkessel properties of the arterial system (i.e., systolic storage and diastolic discharge of the arterial ‘compliance chamber’), and an ‘excess pressure’ thought to arise from wave phenomena (Figure 9). However, this concept received considerable criticism and has since been revised. For example, a key implication of this original hybrid model was that wave intensity analysis should be performed using excess pressure rather than the measured pressure (Wang et al., 2003; Davies et al., 2007; Tyberg et al., 2009). However, subsequent modeling studies, supported by *in vivo* data from both animals and humans, revealed significant problems with this approach (Mynard et al., 2012, 2015; Mynard, 2013; Mynard and Smolich, 2014a). In addition, the assumption that the windkessel pressure was spatially homogenous in the large arterial network was questioned because it exhibited wave-like behavior when measured at multiple locations (Mynard et al., 2012). Others have also raised similar theoretical concerns (Vermeersch et al., 2009; Segers et al., 2015; Westerhof and Westerhof, 2015).

In response to some of the criticisms raised, Parker et al. proposed a ‘modified’ reservoir pressure (*P*_res_) and revised concepts around how this reservoir pressure relates to waves (Parker et al., 2012; Parker, 2017; Hughes and Parker, 2020). The new *P*_res_ is distinct from a windkessel pressure in that it incorporates a propagation delay but is otherwise uniform in space. Moreover, rather than being considered exclusive to wave reflection, it is now broadly agreed that the *P*_res_ in fact arises entirely from wave reflection, with Hughes and Parker (2020) stating that “the reservoir pressure can be understood as the pressure due to the cumulative effect of … reflected and re-reflected waves.” Indeed, it has been shown that in the aorta, *P*_res_ is approximately equal to twice the backward component of pressure (Hametner et al., 2014), while excess pressure (*P*_ex_ = *P* − *P*_res_) is approximately equal to flow multiplied by characteristic impedance, which is the pressure that would theoretically exist in the absence of any wave reflection. The debate regarding wave intensity analysis appears to have been resolved, with recent literature reflecting a consensus that measured pressure (or an appropriate distension-based surrogate) should be used for wave intensity, not *P*_ex_ (Segers et al., 2017; Su et al., 2017; Pomella et al., 2018; Bhuva et al., 2019; Chiesa et al., 2019; Kowalski et al., 2019b; Hughes et al., 2020).

Finally, although aortic *P*_res_ provides similar information to conventional wave separation analysis (since *P*_res_ ≈ 2*P*_–_), a potential benefit of *P*_res_ is that it can be obtained (with several assumptions) from a peripheral site without having to estimate central pressure or flow (Aguado-Sierra et al., 2008; Peng et al., 2017). However, a key consideration when calculating *P*_res_ is that it relies on fitting an exponential curve to diastolic pressure. Vermeersch et al. (2009) found this fitting to be unreliable in 15% of recordings from a cohort of 35–55 year-old adults, and noted that this might be expected when pressure waveforms do not exhibit a clear exponential decay (such as C-type waveforms often observed in younger individuals).

In summary, the reservoir-wave paradigm has seen both significant debate and new developments over the past decade. Further work is needed to determine whether *P*_res_ and *P*_ex_ provide true added value (in both a conceptual and pragmatic sense) over other non-invasive wave analysis techniques, as well as establishing which measurement techniques and in which patient settings it can be reliably applied.

## Closing Remarks

The analysis of waves in blood vessels provides important insights into biomechanical processes and interactions in the cardiovascular system, many of which are not discernable from standard indices such as systolic and diastolic pressure, or cardiac output. Indices obtained from these analyses are therefore extremely useful in a research setting for answering mechanistic questions about cardiovascular physiology in health and disease. There is also emerging evidence that these indices could prove to be valuable in the clinical setting, with the higher specificity of information obtained potentially aiding the goal of personalized medicine (Chirinos, 2017). These wave analysis techniques have not yet been widely adopted in medical practice partly due to the need for further evidence of clinical value. However, other significant barriers likely include a lack of general recognition of the insights that these indices could provide, the relative complexity of measuring and analyzing waves, and the paucity of devices for obtaining wave-related indices in a convenient manner. These barriers constitute fertile ground for future work.

## Author Contributions

JM, AK, and RK drafted the manuscript. JM prepared the figures and tables. JM, AK, RK, MC, and JS edited the manuscript for intellectual content and approved the final version. All authors contributed to the article and approved the submitted version.

## Conflict of Interest

The authors declare that the research was conducted in the absence of any commercial or financial relationships that could be construed as a potential conflict of interest.
